# Actin polymerization inhibition by targeting ARPC2 affects intestinal stem cell homeostasis

**DOI:** 10.1093/burnst/tkad038

**Published:** 2023-10-16

**Authors:** Ruzhen Zhang, Sheng Chen, Zhifan Yang, Ning Zhang, Kenan Guo, Keyi Lv, Zimo Zhou, Meijiao Gao, Xiancheng Hu, Yongping Su, Jianming He, Fengchao Wang

**Affiliations:** State Key Laboratory of Trauma, Burns and Combined Injury, Institute of Combined Injury, Chongqing Engineering Research Center for Nanomedicine, College of Preventive Medicine, Third Military Medical University, Chongqing 400038, China; College of Life Sciences, Chongqing Normal University，Chongqing, 401331 China; State Key Laboratory of Trauma, Burns and Combined Injury, Institute of Combined Injury, Chongqing Engineering Research Center for Nanomedicine, College of Preventive Medicine, Third Military Medical University, Chongqing 400038, China; State Key Laboratory of Trauma, Burns and Combined Injury, Institute of Combined Injury, Chongqing Engineering Research Center for Nanomedicine, College of Preventive Medicine, Third Military Medical University, Chongqing 400038, China; State Key Laboratory of Trauma, Burns and Combined Injury, Institute of Combined Injury, Chongqing Engineering Research Center for Nanomedicine, College of Preventive Medicine, Third Military Medical University, Chongqing 400038, China; State Key Laboratory of Trauma, Burns and Combined Injury, Institute of Combined Injury, Chongqing Engineering Research Center for Nanomedicine, College of Preventive Medicine, Third Military Medical University, Chongqing 400038, China; Department of Laboratory Animal Science, College of Basic Medical Sciences, Army Medical University, Third Military Medical University, Chongqing 400038, China; Department of Military Cognitive Psychology, School of Psychology, Third Military Medical University, Chongqing, 400038, China; State Key Laboratory of Trauma, Burns and Combined Injury, Institute of Combined Injury, Chongqing Engineering Research Center for Nanomedicine, College of Preventive Medicine, Third Military Medical University, Chongqing 400038, China; State Key Laboratory of Trauma, Burns and Combined Injury, Institute of Combined Injury, Chongqing Engineering Research Center for Nanomedicine, College of Preventive Medicine, Third Military Medical University, Chongqing 400038, China; College of Life Sciences, Chongqing Normal University，Chongqing, 401331 China; State Key Laboratory of Trauma, Burns and Combined Injury, Institute of Combined Injury, Chongqing Engineering Research Center for Nanomedicine, College of Preventive Medicine, Third Military Medical University, Chongqing 400038, China; Department of Radiotherapy, Hebei Province Hospital of Chinese Medicine, Hebei University of Chinese Medicine, Key Laboratory of Integrated Chinese and Western Medicine for Gastroenterology Research (Hebei), Shijiazhuang, 050011; State Key Laboratory of Trauma, Burns and Combined Injury, Institute of Combined Injury, Chongqing Engineering Research Center for Nanomedicine, College of Preventive Medicine, Third Military Medical University, Chongqing 400038, China

**Keywords:** F-actin, Benproperine, ARPC2, Intestinal stem cell, YAP

## Abstract

**Background:**

The rapid turnover of the intestinal epithelium is driven by the proliferation and differentiation of intestinal stem cells (ISCs). The dynamics of the F-actin cytoskeleton are critical for maintaining intercellular force and the signal transduction network. However, it remains unclear how direct interference with actin polymerization impacts ISC homeostasis. This study aims to reveal the regulatory effects of the F-actin cytoskeleton on the homeostasis of intestinal epithelium, as well as the potential risks of benproperine (BPP) as an anti-tumor drug.

**Methods:**

Phalloidin fluorescence staining was utilized to test F-actin polymerization. Flow cytometry and IHC staining were employed to discriminate different types of intestinal epithelial cells. Cell proliferation was assessed through bromo-deoxyuridine (BrdU) and 5-ethynyl-2′-deoxyuridine (EdU) incorporation assays. The proliferation and differentiation of intestinal stem cells were replicated *in vitro* through organoid culture. Epithelial migration was evaluated through BrdU pulse labeling and chasing in mice.

**Results:**

The F-actin content was observed to significantly increase as crypt cells migrated into the villus region. Additionally, actin polymerization in secretory cells, especially in Paneth cells (PCs), was much higher than that in neighboring ISCs. Treatment with the newly identified actin-related protein 2/3 complex subunit 2 (ARPC2) inhibitor BPP led to a dose-dependent increase or inhibition of intestinal organoid growth *in vitro* and crypt cell proliferation *in vivo*. Compared with the vehicle group, BPP treatment decreased the expression of Lgr5 ISC feature genes *in vivo* and in organoid culture. Meanwhile, PC differentiation derived from ISCs and progenitors was decreased by inhibition of F-actin polymerization. Mechanistically, BPP-induced actin polymerization inhibition may activate the Yes1-associated transcriptional regulator pathway, which affects ISC proliferation and differentiation. Accordingly, BPP treatment affected intestinal epithelial cell migration in a dose-dependent manner.

**Conclusion:**

Our findings indicate that the regulation of cytoskeleton reorganization can affect ISC homeostasis. In addition, inhibiting ARPC2 with the Food and Drug Administration-approved drug BPP represents a novel approach to influencing the turnover of intestinal epithelial cells.

## Highlights

- Intestinal epithelial cell differentiation was accompanied by an increase in F-actin polymerization.
- Inhibition of F-actin polymerization affects ISC proliferation.
- Cytoskeleton rearrangement interferes with the differentiation trajectory of ISCs.
- Benproperine treatment interferes with intestinal epithelial migration in a dose-dependent manner.

## Background

The small intestine is lined with a simple columnar epithelium that is organized into numerous crypt–villus units. Villi are finger-like protrusions composed of postmitotic cells. Each villus is surrounded by multiple epithelial invaginations known as crypts, where cell division occurs. Lgr5+ intestinal stem cells (ISCs) are located at the base of crypts and actively cycle, with their descendants proliferating and differentiating into secretory and absorptive cells [1]. Most differentiated cells migrate upwards and undergo anoikis at the top of the villi. In contrast, Paneth cells (PCs) are located at the base of the crypt, creating a niche where they surround and support Lgr5 ISCs. [2]. The proliferation and differentiation of ISCs are tightly regulated by both endogenous and exogenous signals to ensure the completion of tissue turnover in 3–5 days with stable cell type ratios [3]. The cytoskeleton, a network of proteins within cells, plays a vital role in mediating cell signal transduction by influencing the strength, localization, and duration of signaling events [4]. Proper regulation of the cytoskeleton is crucial for ISC functions such as proliferation, differentiation, and migration [5–8]. However, the specific impact of cytoskeletal dynamics on ISC homeostasis has yet to be fully understood.

Manipulation of the cytoskeleton has been shown to impact the differentiation of various types of stem cells. For instance, disruption of the actin cytoskeleton using cytochalasin D has been found to enhance adipocyte differentiation in human stromal stem cells, while depolymerizing the cytoskeleton with latrunculin A promotes direct pancreatic differentiation of human pluripotent stem cells *in vitro* [7,9]. Additionally, conditional knockout of the F-actin capping protein CAPZ altered liver physiology, resulting in defects in cell differentiation and organ overgrowth *in vivo* [5]. Overall, the orchestration of mechanical forces from both external [extracellular matrix (ECM) stiffness] and internal (cytoskeleton tension) factors can efficiently regulate stem cell differentiation. Therefore, regulating the balance of internal cytoskeleton organization through small molecule inhibitors is hypothesized to impact cell fate decisions in the intestinal epithelium.

Actin, as the core component of the cytoskeleton, is responsible for a variety of important cellular physiological events [5,10–12]. The actin-related protein-2/3 (ARP2/3) complex plays a pivotal role in both the initiation of actin polymerization and the organization of the resulting filaments. ARP2/3 consists of seven proteins, including two actin-related proteins (ARP2 and ARP3) and five ARP2/3 complex subunits (ARPC1, ARPC2, ARPC3, ARPC4 and ARPC5) [13]. Inhibition of ARP2/3 has recently gained attention due to its association with attenuation of cancer progression, neurotoxic effects during drug abuse and pathogen invasion of host cells [14]. Several small-molecule inhibitors of the ARP2/3 complex have been developed to inhibit actin filament nucleation, including 2-fluoro-*N*-[2-(2-methyl-1*H*-indol-3-yl)ethyl] benzamide (CK666) and C_17_H_16_BrNO_3_S (CK869) [15]. Recently, benproperine phosphate (BPP), which is used as an antitussive drug in clinical settings with unknown mechanisms, was identified as a specific ARPC2 inhibitor that can suppress cancer cell migration and tumor metastasis [16].

In the current study, we first investigated the change in actin polymerization along with intestinal cell differentiation. Then, we utilized the ARPC2 inhibitor BPP to modulate actin polymerization and observed its effect on the proliferation and differentiation of ISCs.

## Methods

### Mice

Male C57BL/6 J wild-type mice, aged 8–10 weeks and weighing 23–24 g, were purchased from GemPharmatech (Chengdu) Co., Ltd. The mice were housed in a specific pathogen-free animal facility with temperature control (22–25°C) and a natural light–dark cycle. All experimental procedures were reviewed and approved by the animal ethics committee of the Third Military Medical University (Chongqing, China).

### Cell line and reagents

MC38, a mouse colon cancer cell line, was purchased from the American Type Culture Collection (Rockville, MD, USA) and grown in DMEM supplemented with 100 ml/l fetal bovine serum under a humidified atmosphere of 5% CO_2_ at 37°C. The other reagents were DMEM/HIGH GLUCOSE (HyClone; SH30022.01), fetal bovine serum (Lonsera; S711–001 s), Western Primary Antibody Dilution Buffer (Beyotime; P0023A), Super ECL Plus Western Blotting Substrate (Baoguang Biotechnology Co. Ltd BG0001), TrypLE™ Express (Thermo; 12 604 013), 10% formalin (Chongqing Chuandong Chemical), paraformaldehyde (Thermo; 305 362), EDTA (Beyotime ST066), nonfat dry milk (Cell Signaling 9999S), goat serum (Bioss; C-0005), TRIzol reagent (Invitrogen; 269 201), bovine serum albumin (BSA) (Beyotime; ST023-200 g), permeabilization buffer 10X (Invitrogen; 2 178 649), advanced D-MEM/F-12 (1X), liquid (Gibco; 12 634–010), IntestiCult™ organoid growth medium (mouse) (STEMCEL; 06000) and penicillin streptomycin solution (100X) (Beyotime; C0222).

### Organoid culture

Mouse crypts were isolated and cultured following previously described methods [17,18]. Briefly, intestines were obtained from 8- to 10-week-old C57BL/6 mice, and the fecal matter was flushed with ice-cold DPBS (HyClone). After longitudinally cutting and washing the intestine with ice-cold DPBS, it was cut into 2–3 mm pieces and incubated in dissociation buffer (DPBS with 30 mM EDTA) for 20 min on ice. After replacing the dissociation buffer with DPBS, pieces of intestine were incubated in a 50 ml tube for another 15 min on ice. After the tube was shaken for 30–60 s, the mixture was passed through a 70 mm cell strainer to isolate and purify crypts via centrifugation at 4°C. Then, crypts mixed with Matrigel (Corning) were seeded in 96-well flat-bottom plates (10 ml/well). The plates were incubated at 37°C for 17 min. Culture medium, IntestiCult™ Organoid Growth Medium (Mouse) (Stem Cell Technologies, Cat: 06005) was added to the wells (100 ml/well). Growth factors [R-spondin 1, epidermal growth factor (EGF) and Noggin] were added every other day and the entire culture medium was replaced after 4 days of seeding. Y-27632(Sigma, Cat:Y0503-1MG) was then withdrawn after 4 days of seeding and not included in subsequent culture maintenance.

### BPP and jasplakinolide treatment

*In vivo*, mice were intraperitoneally injected with BPP (MCE 19428–14-9) at concentrations of 10, 25, 50 or 100 mg/kg daily for three consecutive days. The body weight and status of the mice were observed and the mice were sacrificed on day 3 of BPP administration.

*In vitro*, intestinal crypts were seeded in 96-well plates containing 100 μl of medium as a 3D culture. After organoids were seeded for 24 h, 10 μl of medium of a graded concentration of BPP or jasplakinolide (JAS) was added into each well and incubated at 37°C for 24 h. The control group received 10 μl of medium only. 3-(4,5-Dimethyl-2-thiazolyl)-2,5-diphenyl-2-*H*-tetrazolium bromide (MTT) was used for the activity assay and 5-ethynyl-2′-deoxyuridine (EdU) was used for the cell proliferation assay. MC38 cells were cultured in 6-well plates containing 2 ml of medium in each well, and 10 μl of medium with graded concentrations of BPP was added to each well. Cells were then incubated at 37°C for 24 h.

### Antibodies

The antibodies used in this study include PE anti-mouse CD24 (BioLegend; 101 808; 1 : 200), PE/Cy7 anti-mouse/human CD44 (BioLegend; 103 030; 1 : 100), FVD (Invitrogen Firable Viability Dye660; 65–0864-14; 1 : 500), Alexa Fluor 647 anti-mouse CD45 (BioLegend; 103 124; 1 : 1000), DAPI (Beyotime; C1006), β-actin (Beyotime; AA128–1; 1 : 5000), goat anti-mouse IgG (H + L) (MULTISCIENCES (LIANKE) BIOTECH, CO., LTD; GAM007; 1:300), anti-rabbit IgG (Cell Signaling Technology; 7074S; 1 : 300), GAPDH (Proteintech; HRP-60004; 1 : 10000), PCNA (Cell Signaling Technology; 2586 T; 1 : 2000), MTT (Beyotime; ST1537-1 g), BeyoClick EdU Cell Proliferation kit with Alexa Fluor 488 (Beyotime; C0071S), bromo-deoxyuridine (BrdU) (BD Pharmingen; 555 627; 1 : 300), Yes1-associated transcriptional regulator (YAP1) (Thermo; RF2213666; 1 : 300), Phalloidin (Beyotime; C2201S 1:300)

### Immunofluorescence

Intestines were collected without fecal content and fixed in zinc formalin fixative for 24 h after mice were euthanized. They were dehydrated and embedded to prepare paraffin sections with a thickness of 3 μm. After dewaxing and antigen repair, slices were blocked in PBS containing 5% goat serum and 0.3% Triton X-100 at room temperature for 2 h. Primary antibodies were incubated at 4°C overnight. After washing three times with PBST, secondary antibodies were incubated at room temperature for 2 h. Finally, the DAPI working solution was incubated for 20 min. Images were collected by fluorescence microscopy.

EdU (10 μM) was added to the culture medium of organoids and incubated at 37°C for 3 h. Then, organoids were fixed in 10% formalin for 40 min. After washing three times, the organoids were permeabilized for 20 min with 0.3% Triton X-100. The BeyoClick™ EdU-488 Cell Proliferation Detection KitB assay was used to detect proliferative cells.

After fixation, organoids were blocked in PBS containing 5% goat serum and permeabilized with 0.3% Triton X-100 at room temperature for 2 h. Primary antibodies were incubated at 4°C overnight and then washed three times for 10 min each time. Secondary antibodies were incubated at room temperature for 2 h and washed three times for 10 min each time. Finally, the DAPI working solution was incubated for 20 min. The steps of MC38 cell slide staining were the same as above, and images were collected by fluorescence microscopy.

### Western blotting

Protein was extracted with RIPA splitting buffer including protease and phosphatase inhibitors. A BCA assay was used for quantitative analysis of protein. Protein denatured at 95°C was loaded for 10% SDS-PAGE and then electrotransferred to PVDF membranes. Membranes were blocked with 5% nonfat dry milk or 5% BSA in TBST for 90 min. Primary antibodies were incubated at 4°C overnight, washed three times with TBST for 10 min each time, incubated with secondary antibodies at room temperature for 90 min and washed again. Immunoreactive bands were visualized using a chemiluminescent solution (1 : 1) and detected using a ChemiDoc imaging system (Bio-Rad).

### Flow cytometry

The small intestine crypt cell suspension was incubated for 30 min at 4°C with an antibody mixture of CD24 and CD44 in a total volume of 200 ml of PBS/2% goat serum. After staining, the cells were centrifuged at 430 g for 5 min on ice. Then, the cells were fixed with 4% paraformaldehyde on ice for 30 min, and after fixation with 0.1% Triton X-100, the cells were permeabilized for 30 min. Next, the cells were stained with Actin-Tracker Green at room temperature for 30 min. Finally, the DAPI working solution was resuspended and incubated for 30 min. Flow cytometry was detected on a Sony MA900 cell sorter.

### Real-time PCR quantification

Total RNA was isolated from crypts using TRIzol reagent. According to the manufacturer’s protocol, cDNA was obtained using the R223–01@Vazyme kit. The Vazyme reaction system was used for real-time RT-PCR. The primer sequences are listed in Table 1, and the expression level of each target gene was standardized to that of GAPDH. The relative expression of RNA was measured by the 2^−△△Ct^ method.

**Table 1:** Primers used in this study

**Species**	**Primer**	**Forward(5′–3′)**	**Reverse(5′–3′)**
Mouse	Lgr5	CGTTGTCAGCCGTGGTCATAGC	GCCCTGCGTTTGAGCACTGAA
Mouse	Olfm4	AAGTGACCTTGTGCCTGCCAC	CACCACGCCACCATGACTACAG
Mouse	Lyz1	GCATGGCGAGCACACTGTCAA	TGCCCTGTTTCTGCTGAAGTCC
Mouse Mouse Mouse Mouse	Muc2 GAPDH Klf4 Fcgbp	AAGTCCGCACCAGGCGTTCTA CAGTGGCAAAGTGGAGATTGTTG GCGAGTCTGACATGGCTGT GGGGGCAGCAAAGACATACT	CCAAGCCAGGAGTGGAGAAGGT TCGCTCCTGGAAGATGGTGAT GTTCCTCACGCCAACGGTTA CAGGGCAGGTGATCTCACA

### Statistical analysis

Statistical analysis was performed using GraphPad/Prism8, and FlowJo V10 was used to analyze flow cytometry data. The *p* value of the datasets was determined by unpaired two-tailed Student’s t test, one-way analysis of Variance (ANOVA) and nonparametric test. The level of significance is indicated by asterisks as follows: ^*^p < 0.05, ^*^^*^p < 0.01, ^*^^*^^*^p < 0.001, ^*^^*^^*^^*^p < 0.0001 throughout the Figures. Error bars are expressed as the mean ± SD. The images were typeset using Adobe Illustrator 2021, and Image-Pro Plus (version 6.0) was used for mean fluorescence intensity statistics.

## Results

### Actin content is increased along with intestinal epithelial cell differentiation

**Figure 1:**
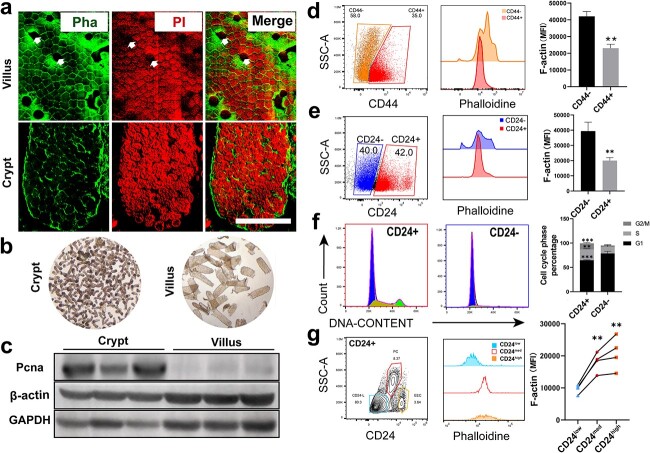
Actin content is increased along with intestinal epithelial cell differentiation. (**a**) Phalloidin (Pha) fluorescence (green) was stained to show F-actin expression in isolated crypt–villus units (scale bar 50 μm; white arrows indicate the absence of phalloidin staining in goblet cells due to their large mucin granules). (**b**) Compartment-enriched villus or crypts. (**c**) F-β-actin expression in crypt cells and villus cells by western blot. (**d**, **e**) F-actin expression in crypt and villus cells and F-actin expression in enriched crypt (CD44+/CD24+) cells and enriched villus (CD44−/CD24−) cells were analyzed by flow cytometry (n = 4; error bars indicate means ± SDs; ^*^^*^p < 0.001 *vs* control). (**f**) The cell cycle in crypt (CD24+) and villus (CD24−) cells was analyzed by flow cytometry (n = 4; error bars indicate means ± SDs; ^*^^*^p < 0.01, ^*^^*^^*^p < 0.001 *vs* control). (**g**) F-actin expression in stem cells and progenitor cells (CD24low: blue gate), Paneth cells (PCs) (CD24med: red gate) and enteroendocrine cells (EECs) (CD24high: yellow gate)was analyzed by flow cytometry. *MFI* mean fluorescence intensity

The small intestine epithelium can be anatomically divided into villi, which are composed of major absorptive cells, and crypts, which are mainly composed of proliferative cells. Villous cells have longer microvilli and more actin-based structures than crypt cells. Thus, much stronger phalloidin fluorescence was found in villi than in crypts (Figure 1a). Compartments enriched in villi or crypts were separated, and western blot (WB) results confirmed the higher expression of β-actin in villous differentiated cells (Figure 1b, c). Flow cytometry data showed that most villous cells were characterized as CD24− and CD44−, while most crypt cells were CD44+ and CD24+ [18]. The average intensity of phalloidin fluorescence in CD44− villous cells was significantly higher than that in CD44+ crypt cells, and the same tendency was found between CD24− villous cells and CD24+ crypt cells (Figure 1d, e). As shown in (Figure 1f), the cell cycle was observed in CD24− villous cells. In contrast, F-actin expression in proliferating cells was relatively lower than that in quiescent cells. Crypt cells contain terminally differentiated cells, including PCs and enteroendocrine cells (EECs). CD24 combined with size scatter was employed to distinguish CD24^med^ PCs and CD24^high^ EECs as previously reported [2]. Interestingly, we found that the F-actin staining in CD24^med^ PCs and CD24^high^ EECs was stronger than that in CD24^low^ ISCs and proliferating progenitor cells (Figure 1g), indicating that cytoskeleton reorganization was immediately increased when ISCs or progenitors differentiated into secretory cells. Taken together, the above data suggest that ISC lineage differentiation couples with the cell cycle and increases cytoskeletal protein expression and reorganization.

### Dose-dependent inhibition of F-actin polymerization affects intestinal epithelial proliferation

**Figure 2:**
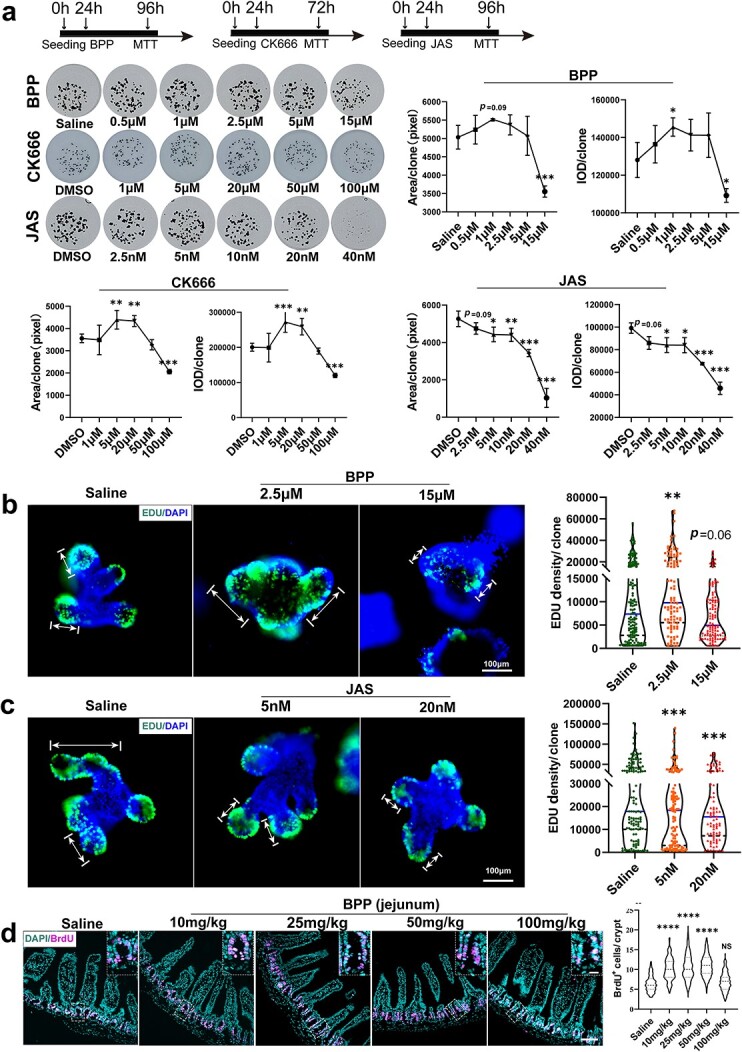
Dose-dependent inhibition of F-actin polymerization affects intestinal epithelial proliferation. (**a**) MTT experiment of organoids treated with different concentrations of BPP, CK666 and JAS; organoid area and integrated optical density (IOD) were measured (n = 3 independent cultures, area > 100 pixels; error bars indicate means ± SDs, one-way ANOVA, ^*^p < 0.05, ^*^^*^p < 0.01, ^*^^*^^*^p < 0.001 *vs* control). (**b**) Organoid proliferation was examined by EdU incorporation for 3 h at 72 h after BPP (0, 2.5 and 10 μM) treatment and mean fluorescence intensity (MFI) statistics for each crypt (scale bar 100 μm; white double arrow: proliferating region, Wilcoxon test, error bars indicate median ± quartile, ^*^^*^p < 0.01 *vs* control). (**c**) Organoid proliferation was examined by EdU incorporation for 3 h at 72 h after JAS (0, 2.5 and 10 μM) treatment and MFI statistics for each crypt (scale bar 100 μm, white double arrow: proliferating region; Wilcoxon test: error bars indicate median ± quartile, ^*^^*^^*^p < 0.001 *vs* control). (**d**) Immunofluorescence staining showed crypt proliferating cells (BrdU^+^) after continuous administration of BPP for 3 days (jejunum) and statistics of the number of BrdU cells per crypt (scale bars 100 and 50 μm, n = 3; error bars indicate means ± SDs, one-way ANOVA, ^*^^*^^*^^*^p < 0.0001 *vs* control). *ANOVA* analysis of Variance, *BPP* benproperine, *JAS* Jasplakinolide, *EdU* 5-Ethynyl-2-deoxyuridine, *BrdU* Bromo-deoxyuridine

To test the effect of manipulating the cytoskeleton on ISC proliferation, a series of F-actin polymerization-regulating small molecular inhibitors were used to treat intestinal organoids. BPP has been identified as a highly specific ARPC2 inhibitor that downregulates the function of the ARP2/3 complex and inhibits G-actin polymerization to restrain lamellipodium formation [16]. Another ARP2/3 complex function inhibitor, CK666, has also been widely used in studies of F-actin inhibition [8,15,19]. JAS, a cyclo-depsipeptide, was reported to be a specific drug to polymerize and stabilize actin filaments [20]. Both the areas and integrated optical density of MTT-stained organoids were assessed to quantify the degree of organoid growth [17]. The results showed that BPP treatment at 0.5, 1 and 2.5 μM obviously promoted the proliferation of organoids, while BPP at 15 μM sharply inhibited growth (Figure 2a). Adding CK666, another F-actin inhibitor, led to similar effects on organoids, promoting proliferation at low doses such as 5 and 20 μM and inhibiting proliferation at high doses such as 100 μM (Figure 2a). However, significant inhibition of organoid growth was observed in the JAS groups at the given doses (Figure 2a). Moreover, EdU labeling was used to evaluate cell proliferation, showing results consistent with the quantification of organoid size-based MTT imaging. The EdU fluorescence density in the 2.5 μM BPP group was significantly higher than that in the saline group but significantly decreased in the 15 μM BPP group (Figure 2b). JAS treatment at different doses decreased the EdU fluorescence density (Figure 2c).

**Figure 3:**
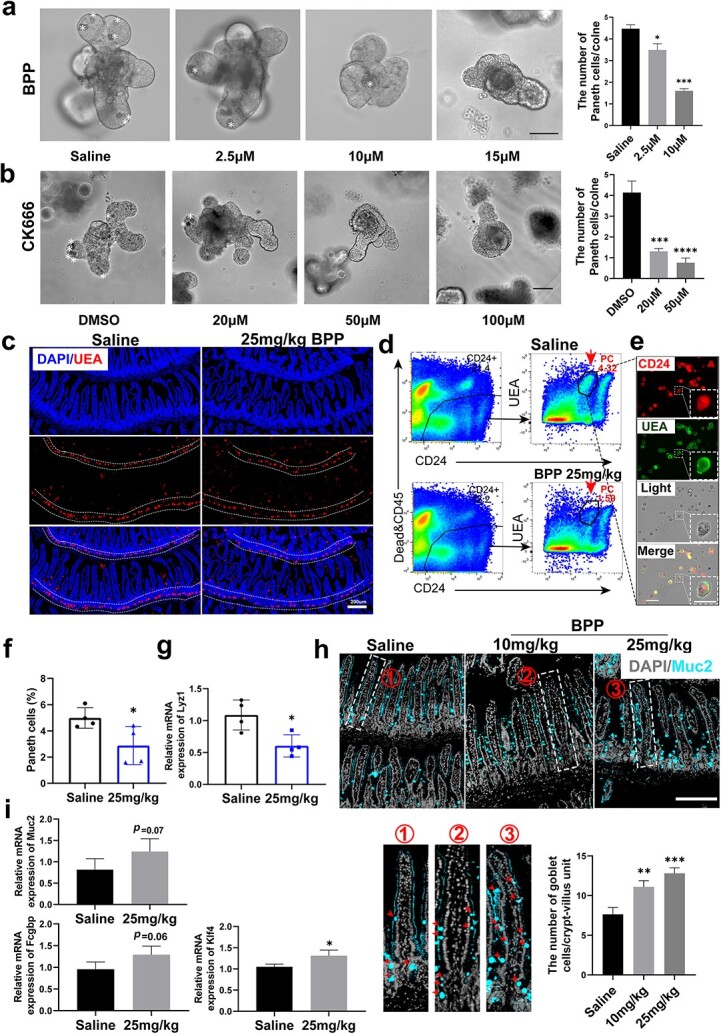
Downregulation of actin polymerization impairs the balance of ISC differentiation into cell lineages. (**a**) Organoids cultured at 0, 2.5, 10 and 15 μM BPP for 3 days (scale bar 100 μm); statistics of the number of PCs per clone (n = 3, independent cultures, one-way ANOVA, error bars indicate means ± SDs, ^*^p < 0.05, ^*^^*^^*^p < 0.001 *vs* control). (**b**) Organoids cultured at DMSO 20, 50 and 100 μM CK666 for 2 days (scale bar = 100 μm); statistics of the number of PCs per clone (n = 4, independent cultures, one-way ANOVA, error bars indicate means ± SDs, ^*^^*^^*^p < 0.001, ^*^^*^^*^^*^p < 0.0001 *vs* control). (**c**) Immunostaining of *Ulex europaeus* agglutinin (UEA) in control and BPP (25 mg/kg administered for three days, scale bar 200 μm). (**d**) Gating strategy for PCs in flow cytometric analysis. (**e**) Immunofluorescence imaging confirmed that the cells in the gate were PCs (scale bar 50 μm). (**f**) Quantification of PCs analyzed by flow cytometry (n = 4, error bars indicate means ± SDs, ^*^p < 0.05 *vs* control). (**g**) Lyz1 mRNA levels measured by RT-qPCR in crypt cells (n = 4, independent-samples t test, error bars indicate means ± SDs, ^*^p < 0.05 *vs* control). (**h**) Muc2 immunostaining in groups adding saline and BPP (0, 10, and 25 mg/kg, one-way ANOVA, error bars indicate means ± SDs, scale bar 200 μm, ^*^^*^p < 0.01, ^*^^*^^*^p < 0.001 *vs* control, red arrows indicate GCs). (**i**) Muc2, Klf4 and Fcgbp mRNA levels measured by RT-qPCR in crypt cells (n = 4, independent-samples t test error bars indicate means ± SDs, ^*^p < 0.05 *vs* control). *ANOVA* analysis of Variance, *ISCs* intestinal stem cells, *PCs* Paneth cells, *BPP* benproperine

**Figure 4:**
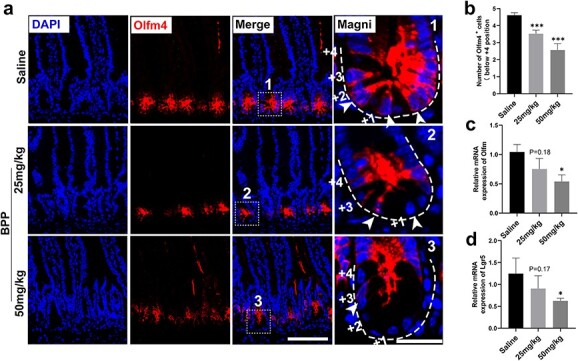
Inhibition of actin polymerization decreases ISC stemness. (**a**) Immunostaining of Olfm4 in crypt cells treated with the indicated doses of BPP (n = 3, scale bar 100 and 25 μm in magnification, white arrows indicate Olfm4+ISCs). (**b**) Statistics of Olfm4-positive cells below the +4 position (n = 3, one-way ANOVA, error bars indicate means ± SDs, ^*^^*^^*^p < 0.001 *vs* control). (**c**, **d**) Olfm4 and Lgr5 mRNA levels measured by RT-qPCR in crypt cells (n = 4, one-way ANOVA, error bars indicate means ± SDs, ^*^p < 0.05 *vs* control). *ANOVA* analysis of Variance, *ISCs* intestinal stem cells, *BPP* benproperine

As BPP has been safely used as an oral medicine, its effect on intestinal crypt cells in mice was tested. BrdU incorporation tests were used to test the proliferation rate in crypt cells. After BPP administration for three continuous days, the number of BrdU+ cells per crypt significantly increased in the BPP group given doses of 10, 25 and 50 mg/kg compared with the saline group. However, the proliferation-promoting effect of BPP *in vivo* disappeared at high doses of 100 mg/kg (Figure 2d). Taken together, these data indicate that inhibiting actin polymerization to a certain extent can promote the proliferation of intestinal crypt cells.

### Downregulation of actin polymerization impairs the balance of ISC differentiation into cell lineages

Considering the dramatic alterations in actin polymerization during differentiation from ISCs or progenitor cells to PCs, we tested the potential effect of ARPC2 inhibition on this differentiation process. The distinctive morphology of PCs, characterized by large secretory granules within their cytoplasm, enables their easy identification in intestinal organoids through differential interference contrast microscopy. The quantification of PCs per organoid revealed a significant number reduction upon treatment with 2.5 μM BPP compared to saline. Moreover, the differentiation of most PCs from ISCs or progenitor cells was inhibited by 10 μM BPP treatment (Figure 3a, b). We also tested the effect of BPP on PC differentiation *in vivo*. Treatment with 25 mg/kg BPP for three continuous days significantly decreased the number of PCs in the crypt base, as evaluated by *Ulex europaeus* agglutinin-positive staining (Figure 3c). In parallel, the number of PCs was quantified using flow cytometry based on CD24+ size scatter [2] (Figure 3d, e). Data from the real-time PCR test showed that the expression of the PC marker gene Lyz1 in crypt cells was significantly decreased (Figure 3f) after BPP treatment (Figure 3g). Meanwhile, goblet cells had relatively lower β-actin content than other differentiated cells (Figure 1a). Interestingly, an obvious increase in the number of goblet cells was observed in the BPP group at doses of 10 or 25 mg/kg compared with the saline group (Figure 3h). Furthermore, real-time PCR showed that the expression of goblet marker genes, including Muc2, Klf4 and Fcgpb, was significantly increased after BPP treatment (Figure 3i). Taken together, these data demonstrate that downregulation of actin polymerization by BPP affects the differentiation of ISCs and progenitor cells and that an increase in actin polymerization is be a requisite for PC differentiation and maturation.

### Inhibition of actin polymerization decreases ISC stemness

Crypt base columnar intestinal stem cells are distinguished by the high expression of Lgr5 and a set of ISC feature genes, including Olfm4 and Ascl2 [21]. To explore the role of actin polymerization in stemness maintenance of Lgr5 ISCs, immunofluorescence staining of Omlf4 was used in different groups. After three continuous days of treatment with 25 or 50 mg/kg BPP, Olfm4-positive cells at the jejunum crypt base were decreased compared with the saline group, especially in the location from positions 0–4, counting from the bottom of the crypt where ISCs are mainly located [22] (Figure 4a, b). Furthermore, the transcriptional levels of Olfm4 and Lgr5 in crypt cells were decreased by BPP treatment *in vivo* (Figure 4c, d). These data indicate that the stemness maintenance of ISCs was sensitive to actin polymerization regulation.

**Figure 5:**
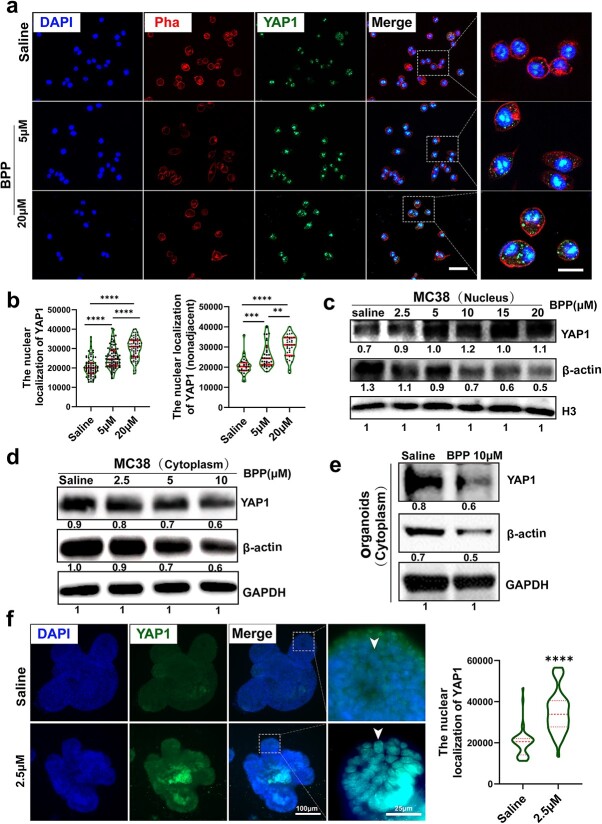
Inhibition of F-actin polymerization enhances YAP1 nuclear localization. (**a**) Immunostaining for YAP1 and phalloidin (Pha) in MC38 cells (harvested after 24 h, scale bars 25 and 10 μm). (**b**) The fluorescence density of YAP1 was quantified in nuclear localization [total cell n = 228 (left); nonadjacent cell n = 97 (right), one-way ANOVA, error bars indicate median ± quartile, ^*^^*^p < 0.01, ^*^^*^^*^p < 0.001, ^*^^*^^*^^*^p < 0.0001 *vs* control]. (**c**) Western blot analysis shows the levels of nucleus YAP1 and β-actin expression after BPP treatment at gradient doses. (**d**) Western blot analysis illustrates the levels of cytoplasm YAP1 and β-actin expression after BPP gradient treatment. (**e**) Western blot analysis illustrates the levels of organoids’ cytoplasm YAP1 and β-actin expression after BPP treatment. (**f**) Immunostaining for YAP1 in gut organoids (n = 3, scale bar 100 and 25 μm, error bars indicate median ± quartile, nonparametric test, ^*^^*^^*^^*^p < 0.0001* vs* control, white arrows indicate localization of YAP1).* ANOVA* analysis of Variance, *YAP1*Yes1-associated transcriptional regulator, *GAPDH*glyceraldehyde-3-phosphate dehydrogenase, *DAPI* 4',6-diamidino-2-phenylindole, *BPP* benproperine

**Figure 6:**
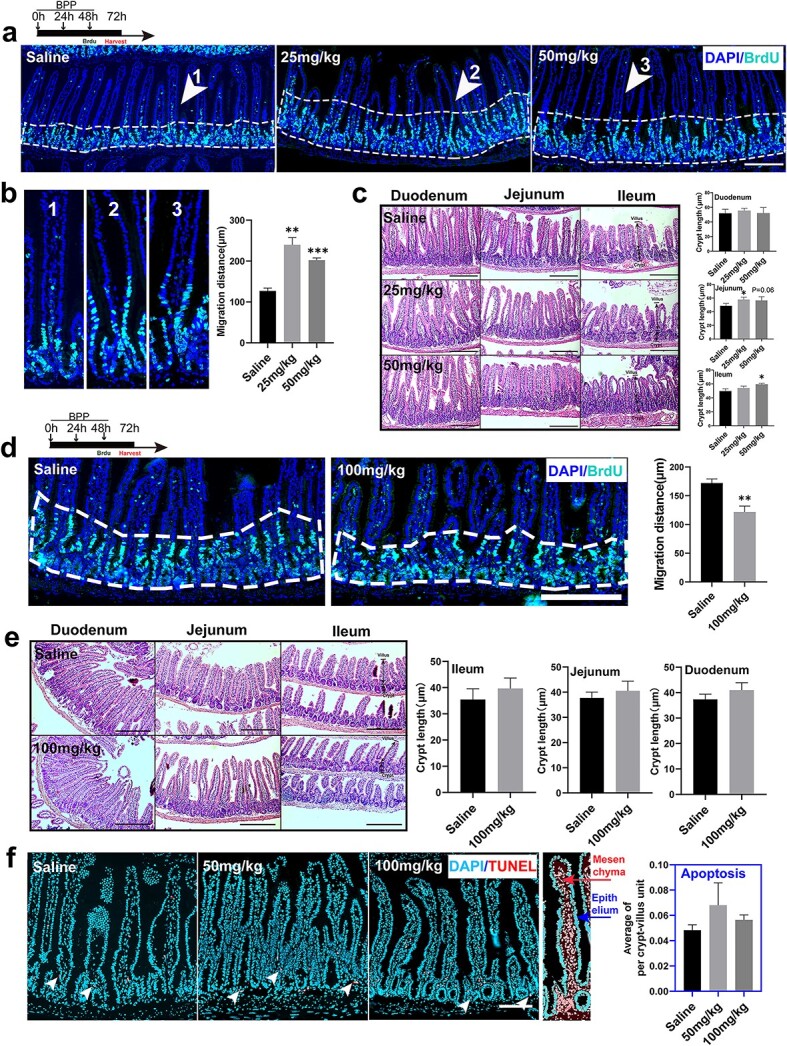
Inhibition of actin polymerization affects intestinal epithelial turnover in a dose-dependent manner. (**a**) BrdU pulse–chase assays (scale bar 200 μm, BPP was administered in low doses). (**b**) Representative crypt–villus unit display and histogram for BrdU fronts (maximum Z projections, n = 4, one-way ANOVA, error bars indicate means ± SDs, scale bar 200 μm, ^*^^*^p < 0.01, ^*^^*^^*^p < 0.001* vs* control). (**c**) Hematoxylin-eosin (HE) staining showed a significant increase in crypts in the BPP group (saline, 25 and 50 mg/kg, administered for 3 days, scale bar = 200 μm) and histogram for the length of crypt (n = 3, one-way ANOVA, error bars indicate means ± SDs, scale bar 200 μm, ^*^p < 0.05* vs* control). (**d**) BrdU pulse–chase assays (BPP was administered in large dose) and histogram for distance of BrdU fronts (maximum Z projections, n = 3 error bars indicate means ± SDs, independent-samples t test, scale bar 200 μm, ^*^^*^p < 0.01* vs* control). (**e**) HE staining showed no significant difference between BPP and the control group (saline, 100 mg/kg, administered for 3 days, scale bar 200 μm) and histogram for the length of crypt (n = 3, one-way ANOVA, error bars indicate means ± SDs, scale bar 200 μm). (**f**) Immunostaining for TUNEL in control and BPP and histogram for TUNEL^+^ cells (saline, 50 and 100 mg/kg BPP was administered for 3 days, one-way ANOVA, means ± SDs, scale bar 200 μm, white arrows indicateepithelial apoptotic cells). *BPP* benproperine, *ANOVA* analysis of Variance

### Inhibition of F-actin polymerization enhances YAP1 nuclear localization

Inhibition of F-actin polymerization resulted in downregulation of the expression of Lgr5+ ISC feature genes and affected their differentiation, particularly increasing differentiation toward PCs. These findings are reminiscent of a recent study that showed that YAP1 activation inhibits Wnt pathway activation and prevents PC differentiation in the early phase of intestinal regeneration after irradiation exposure [23]. Hence, the effect of actin polymerization on YAP1 was explored by using a mouse intestinal tumor-derived cell line MC38 and organoids. Immunofluorescence staining of YAP1 in 2D cultures showed that YAP1 nuclear localization was significantly increased in the BPP groups compared with the vehicle group (Figure 5a, b). To further quantify YAP1 nuclear translocation, nuclear protein of MC38 was extracted and assayed using WB. The results showed that treatment with 2.5 μM or higher doses of BPP increased the level of YAP1 in the nucleus (Figure 5c), while YAP1 expression in the cytoplasm was significantly reduced (Figure 5d). Similarly, after BPP treatment, a significant increase in YAP1 nuclear immunofluorescence staining was observed in the organoid budding area where ISCs were located (Figure 5f). Accordingly, a decreased level of YAP1 in the cytoplasm was observed in organoid samples (Figure 5e). Together, these data indicate that inhibition of F-actin polymerization by targeting ARPC2 activates the YAP pathway in intestinal epithelial cells, which may contribute to regulating ISC proliferation and differentiation.

### Inhibition of actin polymerization affects intestinal epithelial turnover in a dose-dependent manner

Intestinal epithelium turnover occurs at a steady speed. The constant cell migration along the crypt–villus axis is driven both by mitotic pressure from crypts and active migration motivated by the ARP2/3 complex [24]. To investigate the effect of BPP on intestinal epithelial cell migration, a BrdU pulse–chase assay was performed in mice injected with BPP at given doses. The measurement of migration distance showed that intestinal epithelial cells moved faster in the BPP groups than in the control group (Figure 6a, b) at doses of 25 and 50 mg/kg, which is consistent with the increased cell proliferation observed in Figure 2d. We further evaluated the changes in crypt length and found that there was a significant increase in crypt length in the jejunum and ileum of mice treated with a low dose of BPP (Figure 6c). This suggests that the increased mitotic pressure induced by low doses of BPP promotes cell migration across the crypt–villus boundary. However, when the dose of BPP was increased to 100 mg/kg, the migration rate of epithelial cells in the BPP groups was considerably slower (0.71 times) than that of the control groups (Figure 6d), and the difference in crypt length became less significant (Figure 6e). This may be due to the decreased cell proliferation in the crypt and the efficient inhibition of active cell migration by targeting the ARP2/3 complex. In addition, to exclude potential cell death caused by high-dose BPP treatment, which may affect cell migration, we performed TUNEL staining. The results showed that even high doses of BBP treatment at 50 and 100 mg/kg did not cause apparent apoptosis in crypt cells (Figure 6f). These data indicate that pharmacological inhibition of actin polymerization may affect the turnover speed of the intestinal epithelium in a dose-dependent manner.

## Discussion

The maintenance of a functional intestinal barrier requires rapid turnover with a relatively steady speed and a proper cell type component. Dysregulation of either the proliferation and differentiation of ISCs or migration of differentiated cells could lead to pathologies, such as cancer and inflammatory diseases [24–26]. Here, we showed that increased F-actin polymerization was accompanied by the differentiation of ISCs and progenitor cells into the most terminally differentiated cells, especially PCs. We found that inhibiting the ARP2/3 complex unit ARPC with the newly identified inhibitor BPP affected the proliferation, differentiation and migration of intestinal crypt cells in a dose-dependent manner. Activation of the YAP pathway may be a potential mechanism underlying these effects.

Intracellular signaling pathways can be modulated by mechanical forces both within and outside the cell [27]. The microenvironment of cells lining the villus finger-like protrusion differs from that of cells in the crypt, which has an invaginated structure. The mechanical forces exerted on villi may play a role in cell maturation and fate determination. Interestingly, PCs located adjacent to Lgr5 ISCs exhibit much higher expression levels of actin and F-actin polymerization than proliferating ISCs and progenitors. A recent study indicated direct differentiation from Lgr5 ISCs to PCs, with a switch from Wnt/β-catenin to noncanonical Wnt/PCP signaling priming cell cycle arrest [28]. In the present study, we found that most postmitotic differentiated cells had higher levels of actin polymerization than proliferating ISCs and progenitor cells. Moreover, actin expression and polymerization changes accompanied cell cycle arrest, cell fate decisions and cell maturation. On the other hand, PCs work as niche cells to support ISCs by secreting important factors [2]. In addition, previous studies have also indicated that direct contact with PCs is critical for Lgr5 ISC colony formation in Matrigel [29]. Thus, it remains unclear whether the stiffness of neighboring PCs is important for maintaining ISC homeostasis. Moreover, mature PCs were reported to dedifferentiate into ISCs after irradiation exposure [30]. Our data suggest that downregulation of actin expression and inhibition of F-actin polymerization are critical steps in initiating the dedifferentiation process.

The lgr5 ISC pool was delicately regulated by multiple signaling pathways, in which the Wnt pathway dominates [3,31]. Activation of the YAP pathway has been shown to decrease Lgr5 feature gene expression [23]. YAP is a transcriptional coactivator downstream of Hippo signaling and mediates the increased expression of genes involved in cell proliferation and differentiation [32–35]. The critical role of YAP in mechanosensing and mechanotransduction has been well recognized [36,37]. Our data indicate that low doses of BPP, an inhibitor of F-actin polymerization, can increase crypt cell proliferation, decrease Olfm4+ ISC number and activate the YAP pathway, which is consistent with recent findings in Olfm4 CreERT-driven Cell division cycle 42 (Cdc42) knockout mice [38]. Cdc42, a member of the Rho guanosine triphosphatase family, is a key regulator of actin dynamics, functioning to connect multiple signals to actin polymerization [39]. Cdc42 knockout in crypts induced a decrease in ISC populations and the expansion of transient amplified cells, accompanied by elevated YAP/TAZ-Ereg signaling activation [38]. Our IF data showed that the Olfm4 signal decreased in the PC zone where Lgr5 ISCs were located and BrdU+ cells increased in the upper crypt where transient amplified cells were located, indicating that targeting Aprc2 can partially copy the phenotype caused by Cdc42 knockout in crypts. In addition, although the specificity of the BPP target on ARPC2 has been thoroughly analyzed in previous studies [16], we cannot completely exclude the off-target effect of BPP on ISCs. Consistently, another widely used ARP2/3 complex inhibitor, CK666, also affected the proliferation and differentiation of ISCs in a dose-dependent manner, which strengthened our hypothesis based on BPP treatment.

ARP2/3-mediated actin cytoskeletal rearrangement plays a critical role in tumor metastasis [11,40]. Thus, ARP2/3 complex inhibitors are gaining attention for cancer treatment [14]. BPP has been widely used as an antitussive drug. Our data indicate that BPP usage may affect intestinal epithelium turnover and explain its gastrointestinal adverse reactions, especially when used at high dosages. It is important to identify the Aprc2 target of BPP and its high efficiency for suppressing cancer cell migration and tumor metastasis. On the other hand, our data imply that caution should be used in applying BBP for some types of cancer, because its proliferation-promoting effect and YAP pathway activation may aggravate the development of tumors and reduce the effect of chemotherapy drugs.

## Conclusions

In summary, our data demonstrate that inhibiting actin polymerization by targeting ARPC2 can affect the proliferation and differentiation of ISCs and progenitor cells. Moreover, new knowledge about the usage of BPP may be allied to gastrointestinal diseases, including colon cancer, inflammatory diseases and tissue damage caused by different types of injuries.

## Data Availability

The data used to support the findings of this study are available from the corresponding author upon request.

## Abbreviations

ARP 2/3: Actin-related protein-2/3; ARPC1: Actin-related protein 2/3 complex subunit 1; ANOVA: Analysis of Variance; BSA: Bovine serum albumin; BrdU: Bromo-deoxyuridine; BPP: Benproperine; CK666: 2-Fluoro-N-[2-(2-methyl-1*H*-indol-3-yl)ethyl] benzamide; CK869: C17H16BrNO3S; DAPI: 4',6-diamidino-2-phenylindole; DMEM: Dulbecco’s Modified Eagle’s Medium; DPBS: Dulbecco's Phosphate-Buffered Saline; ECM:Extracellular Matrix; EdU: 5-Ethynyl-2′-deoxyuridine; EGF: epidermal growth factor; EECs: enteroendocrine cell; GAPDH: glyceraldehyde-3-phosphate dehydrogenase; IF: Immunofluorescence; IHC: Immunohistochemistry; ISCs: Intestinal stem cells; JAS: Jasplakinolide; MTT: 3-(4,5-Dimethyl-2-thiazolyl)-2,5-diphenyl-2-*H*-tetrazolium bromide; PBS:phosphate buffer; PC: Paneth cell; PCNA:Proliferating Cell Nuclear Antigen; WB: Western blot; TUNEL: TdT-mediated dUTP nick end labeling; YAP1: Yes1-associated transcriptional regulator.
